# The Effect of Iron Oxide Insertion on the In Vitro Bioactivity, and Antibacterial Properties of the 45S5 Bioactive Glass

**DOI:** 10.3390/biomimetics9060325

**Published:** 2024-05-29

**Authors:** Imen Hammami, Suresh Kumar Jakka, Isabel Sá-Nogueira, João Paulo Borges, Manuel Pedro Fernandes Graça

**Affiliations:** 1 I3N and Physics Department, Aveiro University, 3810-193 Aveiro, Portugal; imenhammami@ua.pt (I.H.); suresh@ua.pt (S.K.J.); 2 Associate Laboratory i4HB—Institute for Health and Bioeconomy, NOVA School of Science and Technology, NOVA University Lisbon, 2819-516 Caparica, Portugal; isn@fct.unl.pt; 3 UCIBIO—Applied Molecular Biosciences Unit, Department of Life Sciences, NOVA School of Science and Technology, NOVA University Lisbon, 2819-516 Caparica, Portugal; 4 I3N-CENIMAT and Materials Science Department, NOVA School of Science and Technology, Campus de Caparica, Nova University Lisbon, 2829-516 Caparica, Portugal; jpb@fct.unl.pt

**Keywords:** Bioglass^®^, iron, antibacterial activity, bone regeneration, osteointegration, bioactivity, tissue engineering

## Abstract

The aging population and increasing incidence of trauma among younger age groups have heightened the increasing demand for reliable implant materials. Effective implant materials must demonstrate rapid osseointegration and strong antibacterial properties to ensure optimal patient outcomes and decrease the chance of implant rejection. This study aims to enhance the bone–implant interface by utilizing 45S5 bioglass modified with various concentrations of Fe_3_O_4_ as a coating material. The effect of the insertion of Fe_3_O_4_ into the bioglass structure was studied using Raman spectroscopy which shows that with the increase in Fe_3_O_4_ concentration, new vibration bands associated with Fe-related structural units appeared within the sample. The bioactivity of the prepared glasses was evaluated using immersion tests in simulated body fluid, revealing the formation of a calcium phosphate-rich layer within 24 h on the samples, indicating their potential for enhanced tissue integration. However, the sample modified with 8 mol% of Fe_3_O_4_ showed low reactivity, developing a calcium phosphate-rich layer within 96 h. All the bioglasses showed antibacterial activity against the *Gram-positive* and *Gram-negative* bacteria. The modified bioglass did not present significant antibacterial properties compared to the bioglass base.

## 1. Introduction

The field of biomedical implants has evolved from having a main emphasis on mechanical characteristics to a more sophisticated understanding that incorporates critical biological qualities. In the past, the mechanical aspects of implants remained crucial for restoring normal tissue functions, with titanium being the predominant material due to its outstanding mechanical properties [1,2]. However, recent findings emphasize the need to integrate biocompatibility, osseointegration, tissue regeneration, and other biological factors as fundamental components of successful implant design [3,4]. The realization that clinical outcomes are intricately linked to the interaction of the implant with the biological fluid has prompted advancements in materials and surface modifications [5,6,7,8]. In recent years, there has been a growing interest in coating implants with bioactive materials to enhance biological performance including biocompatibility, osseointegration, and tissue regeneration, therefore ensuring enhanced longevity, stability, and overall success in clinical applications [7,9,10].

One of these bioactive materials is the 45S5 bioglass^®^ (45% SiO_2_, 24.5% Na_2_O, 24.5% CaO, and 6% P_2_O_5_ (wt)), invented by L.L. Hench, which has emerged as a compelling class of ceramic materials, notably for bone regeneration [11,12,13]. Their innate bioactivity, characterized by the capacity to form a robust and direct bond with tissues through subsequent chemical reactions, makes them promising candidates for various medical applications [14,15,16]. Beyond their efficacy in stimulating bone growth, bioglasses exhibit intrinsic antibacterial properties, a feature that has attracted significant attention in recent research [17,18]. Notably, this antibacterial activity is not dependent on the inclusion of ions to their composition but arises from factors such as localized pH elevation caused by the exchange of glass network-modifier ions with H^+^ ions from surrounding fluids and osmotic pressure increases [19].

Researchers have explored the incorporation of therapeutic metal ions into bioglass to confer additional biological properties, including improved antibacterial performance, osteogenesis, and anti-inflammatory properties [7,20,21,22,23,24,25,26]. Iron (Fe) has demonstrated exceptional performance in numerous biomedical applications and is considered a promising candidate due to its chemical stability, biocompatibility, and resistance to corrosion [27]. Additionally, it is involved in bone metabolism, particularly in the processes of osteoblastic differentiation, proliferation, and calcification [28]. Furthermore, recent research investigates the use of iron nanoparticles in cancer treatment, exploiting their magnetic properties for targeted drug delivery and hyperthermia treatment [29]. Moreover, studies have demonstrated the antibacterial effects of iron oxide against several types of bacteria, thereby inhibiting biofilm formation [29,30].

The electrical characteristics of bioglass, particularly its capacity to store electrical charges, have been investigated for their potential influence on osseointegration [31,32,33]. The charge storage capacity of bioglass is attributed to the high mobility of the sodium ion chain. Because of its distinctive chemical composition, bioglass can effectively store and release electrical charges, with potential therapeutic application. In their research, Obata et al. [32,33] showed that the bioglass was able to store larger electrical charges compared to hydroxyapatite (HA). Specifically, the calculated stored electrical charges of the bioglass were found to be 1000 times greater than those of HA. In another study, Obata et al. [31] demonstrated that the presence of surface charges on bioglass improves its osteoconductivity by controlling the growth of calcium phosphate crystals. Moreover, Singh et al. [34] observed in their work an increased growth rate of osteoblast cells on negatively polarized 1393B3 bioglass (56% B_2_O_3_, 5.5% Na_2_O, 18.5% CaO, 11.1% K_2_O, 3.7% P_2_O_5_, 4.6% MgO (wt.%)). The enhanced cellular response suggests that surface polarization has a positive effect on the biological performance of the bioglass.

In this study, 45S5 bioglasses modified with the insertion of various percentages of iron oxide (Fe_3_O_4_) were prepared using the melt-quenching method. While the incorporation of hematite Fe_2_O_3_ into the bioglass system has been thoroughly explored, the insertion and effect of magnetite Fe_3_O_4_ in the preparation of the 45S5 bioglass is not widely documented in the literature. The influence of Fe_3_O_4_ on the bioglass structure was evaluated using Raman spectroscopy. Impedance spectroscopy (IS) was employed to validate alterations in the electrical properties of the prepared glasses. To assess the bioactivity of the prepared samples, an immersion test in simulated body fluid (SBF) was used. The antibacterial properties of the bioglasses were analyzed by examining their ability to hinder the growth of *S. aureus* bacteria, a common source of implant infections, as well as other bacterial strains known to contribute to the formation of detrimental biofilms.

## 2. Materials and Methods

### 2.1. Materials Synthesis

The melt-quenching method was employed to fabricate the bioglass, following the composition of 45S5 Bioglass^®^ (46.1SiO_2_-24.4Na_2_O-26.9CaO-2.6P_2_O_5_, mol%) as outlined by Hench et al. [11,12]. Various concentrations of iron oxide (Fe_3_O_4_) (0, 1, 2, 4, and 8 mol%) were introduced to the bioglass network (denoted as Fe0, Fe1, Fe2, Fe4, and Fe8 respectively). The planetary ball-milling technique was employed for 1 h at 300 rpm to mix and homogenize the chemical precursors, namely SiO_2_, P_2_O_5_, CaCO_3_, Na_2_CO_3_, and Fe_3_O_4_. These precursors were supplied by Sigma-Aldrich in Darmstadt, Germany, with a high purity grade of ≥99%. Then, the mixed powder underwent a calcination process at 800 °C for 1h followed by a melting process at 1300 °C for 1 h. To ensure greater sample homogeneity, the bioactive glass was re-melted using the same conditions. The resulting bulk samples from the melt-quenching process were manually ground and subsequently milled in a planetary ball mill system for 1 h at 300 rpm. A series of bioglass compositions (mol%): (100 − x) 45S5 bioglass + x Fe_3_O_4_ is obtained. The nominal composition of the prepared bioglass samples is described in Table 1.

### 2.2. Structural Characterization

Raman spectroscopy was performed on the bulk materials using a Jobin Yvon HR800 spectrometer, equipped with an Ar^+^ laser (λ = 532 nm). The spectra were obtained in back-scattering geometry, encompassing the spectral range of 200 to 1400 cm^−1^.

The XRD diffractograms were obtained at room temperature on an Aeris-Panalytical diffractometer using CuK_α_ radiation (λ = 1.54056 Å). The measurement parameters were a 2θ range of 10°–70° and a scan step of 0.002°.

### 2.3. Electrical Characterization

Electrical measurements were carried out on glass bulk samples having a consistent thickness of 1 mm. Utilizing ImageJ software 1.8.0, the surface area of the samples was determined. The samples were then coated with silver conducting paste on their opposite parallel sides. A nitrogen bath cryostat was employed for alternating current (AC) investigations, enabling temperature measurements ranging from 100 to 400 K. The temperature of the sample was regulated using an Oxford Research IT-C4 system equipped with a platinum sensor for temperature monitoring. To assess dielectric properties in AC experiments, an impedance analyzer (Agilent 4294A, Santa Clara, CA, USA) was utilized. Measurements were performed using the C_p_−R_p_ configuration and a 0.5 V AC signal across a frequency range spanning from 100 Hz to 1 MHz. Using Equations (1) and (2), the complex electric permittivity ε* and the complex dielectric modulus M* were calculated [35,36,37,38,39].

ε* = ε′ − j ε″ = C_p_ (d/ε_0_ A) − j d (ω R_p_ ε_0_ A), (1)

M* = 1/ε* = M′ + j M″ = ε′/(ε′^2^ + ε″^2^) + j ε″/(ε′^2^ + ε″^2^), (2)

where C_p_ and R_p_ are the measured capacitance and resistance, d is the sample thickness, A the electrode area, ω is the angular frequency, and ε_0_ the permittivity of the space (8.8542 × 10^−12^ F/m).

To calculate the activation energy, the Arrhenius model was employed to adjust the temperature dependence of the relaxation frequency [35,37,40,41,42]:

f_max_ = f_0_ exp (−E_a_/(k_B_ T)), (3)

where f_max_ is the frequency where the M″ reaches a maximum for a specific temperature, f_0_ is a preexponential factor, E_a_ is the activation energy, K_B_ is the Boltzmann constant, and T is the temperature.

### 2.4. Bioactivity

Bioactivity test was carried out in accordance with ISO23317:2017 Standards: “Implants for surgery—In vitro evaluation for apatite-forming ability of implant materials” [43]. For the experiment, pellets made from bioactive glass powder with a diameter of 7 mm were immersed in simulated body fluid (SBF) and placed in an incubator set at 37 °C, equipped with a continuous oscillating support. It is crucial to note that the SBF solution was renewed every 48 h to replicate the biological environment accurately. At intervals of 12, 24, 48, 96 h, 14, and 28 days of SBF immersion, the samples were removed from the medium, rinsed with deionized water, and left to dry at room temperature. The surface of the samples was examined using SEM (Scanning Electron Microscopy), a microscope from TESCAN model Vega 3 (TESCAN ORSAY HOLDING, Brno-Kohoutovice, Czech Republic), equipped with Bruker EDS (energy dispersive spectroscopy) system. Additionally, the pH of the SBF medium was evaluated in both the group of samples that underwent medium changes and the group that remained unchanged. The test was conducted in duplicate.

### 2.5. Antibacterial Activity

Agar diffusion assay plates were utilized to observe the antimicrobial behavior of all the samples. Reference strains used were *Escherichia coli* K12 DSM498 (DSMZ, Braunschweig, Germany), *Staphylococcus aureus* COL MRSA (a methicillin-resistant strain provided by Rockefeller University), and *Streptococcus mutans* DSM20523 (DSMZ, Braunschweig, Germany). The bacterial strains were cultivated in tryptic soy broth (TSB) at 37 °C overnight. Prior to testing, the disks with a diameter of 6 mm were sterilized at 180 °C for 2 h. The bioassay consisted of two layers: a base layer solidified with agar 1.5% *w*/*v*, and a top layer at 0.8% *w*/*v*. Plates were prepared with a base layer of 18–20 mL and an overlay of 4 mL of molten seeded bacteria containing around 10^8^ CFU/mL of the relevant indicator bacteria. The disks of material to be tested were placed in the center of the plates, and then placed in an incubator at 37 °C for 24 h. For *S. mutans*, an incubator with 5% CO_2_ was utilized [14].

Photographs of the pellets were captured, and the diameter of the resulting inhibition zone was assessed using ImageJ software. To ensure precision, each pellet underwent thorough measurement from various angles, with a total of 30 measurements per pellet. Statistical analysis of the data was conducted using GraphPad Prism 8.0 software, utilizing an unpaired t-test, to compare the antibacterial effects of the bioactive glass base composition with each of the different samples.

## 3. Results and Discussion 

### 3.1. Structural Characterization

**Figure 1:**
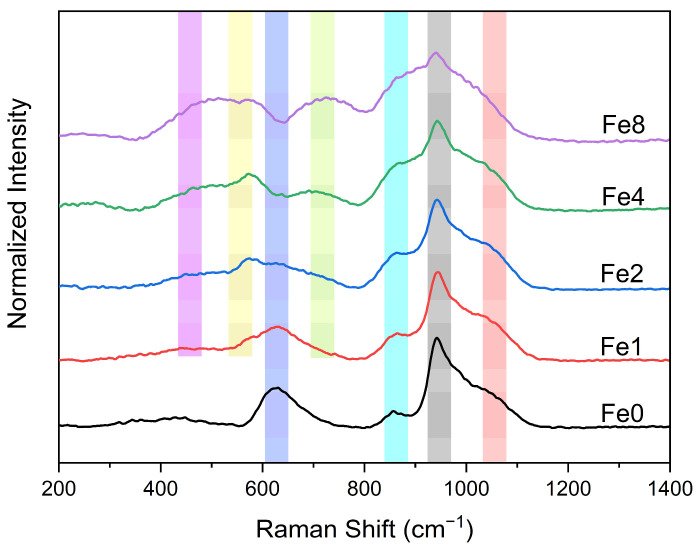
Raman spectra of the various bioglasses.

The structural spectroscopic characteristics of the bioglass samples are analyzed by Raman spectroscopy, as depicted in Figure 1. One can notice that the insertion of iron oxide alters the glass structure. In the low-frequency region (>800 cm^−1^), the bioglass base, Fe0, exhibits a band at around 630 cm^−1^ attributed to the rocking motion of bridging oxygen in structural units containing non-bridging oxygen ions (NBOs) [44,45,46]. However, with an increase in the concentration of Fe_3_O_4_, the intensity of this band decreases, and additional bands are observed. The appearance of the band at around 730 cm^−1^ in the spectra of the iron-containing glasses is likely attributed to the introduction of Fe_3_O_4_, which results in the formation of Fe-related structural units within the sample. A similar feature has been observed in the Raman spectra of nano-Fe_3_O_4_ samples and silicate glasses doped with Fe_3_O_4_ [47,48]. Moreover, the presence of two bands at 560 cm^−1^, and 480 cm^−1^, with intensities increasing as the concentration of Fe_3_O_4_ inserted in the bioglass increases, matches well with the vibrational modes of hematite [49,50].

**Figure 2:**
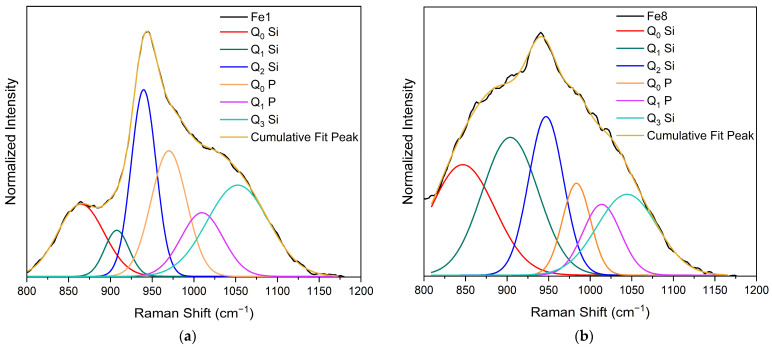
Deconvoluted Raman spectra of (**a**) Fe1 and (**b**) Fe8 samples.

In the high-frequency region (<800 cm^−1^), three vibrational bands can be observed at around 850 cm^−1^, 940 cm^−1^, and 1050 cm^−1^. This region is considered significant as it contains the most intriguing vibrational modes for silicate glasses, characterized by symmetric and asymmetric stretching. Consequently, a deconvolution of the Raman spectra within this region was conducted. The spectral deconvolutions for the bioglass modified with 1, and 8% mol of Fe_3_O_4_ are illustrated in Figure 2a and Figure 2b, respectively. The results show the presence of six vibrational modes located at around 845–860 cm^−1^, 905–908 cm^−1^, 940–906 cm^−1^, 970–985 cm^−1^, 1009–1014 cm^−1^, and 144–1053 cm^−1^, associated with Q_0_ Si units, Q_1_ Si units, Q_2_ Si units, Q_0_ P units, Q_1_ P units and Q_3_ Si units, respectively [44,51,52,53,54,55].

### 3.2. Electrical Characterization

**Figure 3:**
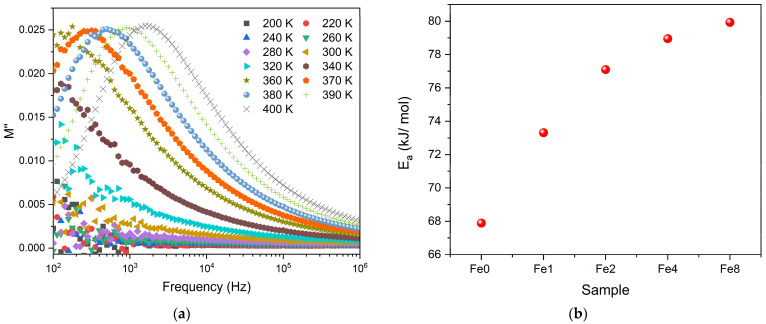
(**a**) The imaginary part of the electrical modulus M″ versus frequency for Fe2 sample and (**b**) the variation activation energy E_a_ with increasing the Fe_3_O_4_ content.

The dielectric properties of the bioglasses were represented in the form of modulus formalism, M*, expressed as 1/ε*. This method offers the advantage of reducing the impact of low capacitance factors, such as electrode polarization and low-frequency conductivity, on the dielectric data. The results, shown in Figure 3a, indicate a single dielectric relaxation process that shifts to higher frequencies with increasing temperature which indicates the presence of a thermally activated process. Notably, other formalisms, such as permittivity, impedance, or admittance, do not clearly show this dielectric relaxation behavior. Therefore, the observed relaxation phenomenon is due to an intrinsic characteristic related to the formation of dipoles between the network modifier ions and the nearby non-bridging oxygen ions. The variation of the relaxation frequency versus temperature determined from M″, was adjusted using the Arrhenius model to calculate the activation energy of this relaxation process. Figure 3b shows that the activation energy E_a_ increases with the increase in Fe_3_O_4_ concentration in the bioglass network. This suggests that the glass structure becomes more rigid, creating higher barriers for ion movement and thereby increasing the activation energy [51].

**Figure 4:**
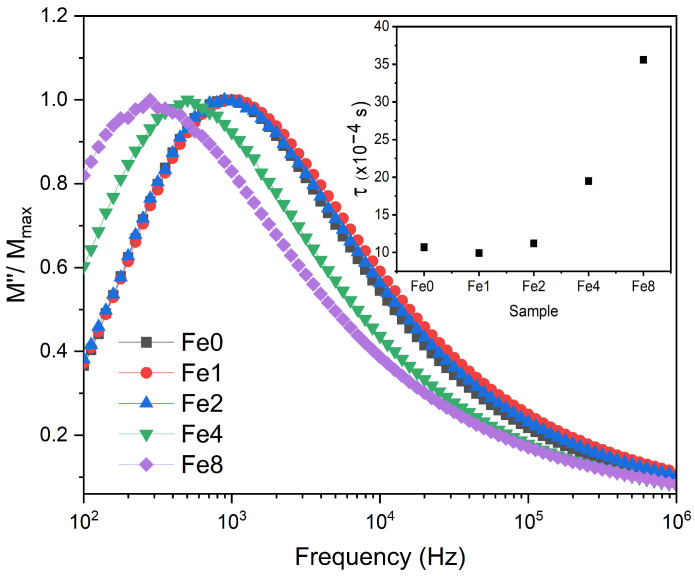
The normalized imaginary part of the modulus M″/M″_max_ versus the frequency at 390 K for all prepared bioglasses (the inset depicts the relaxation time for all samples at 390 K).

Figure 4 displays a comparison of the normalized imaginary parts of the electric modulus M″/M″_max_ versus frequency for different concentrations of Fe_3_O_4_ incorporated into the Bioglass network. A shift in the electrical modulus to a lower frequency was observed with the insertion of a high concentration of Fe_3_O_4_ suggesting an increase in relaxation time (inset of Figure 4). This suggests that the dipoles in the bioglass system have less freedom to align with the direction of the applied electric field. Consequently, the network of bioglasses modified with more than 2 mol% Fe_3_O_4_ is more polymerized.

### 3.3. Bioactivity

**Figure 5:**
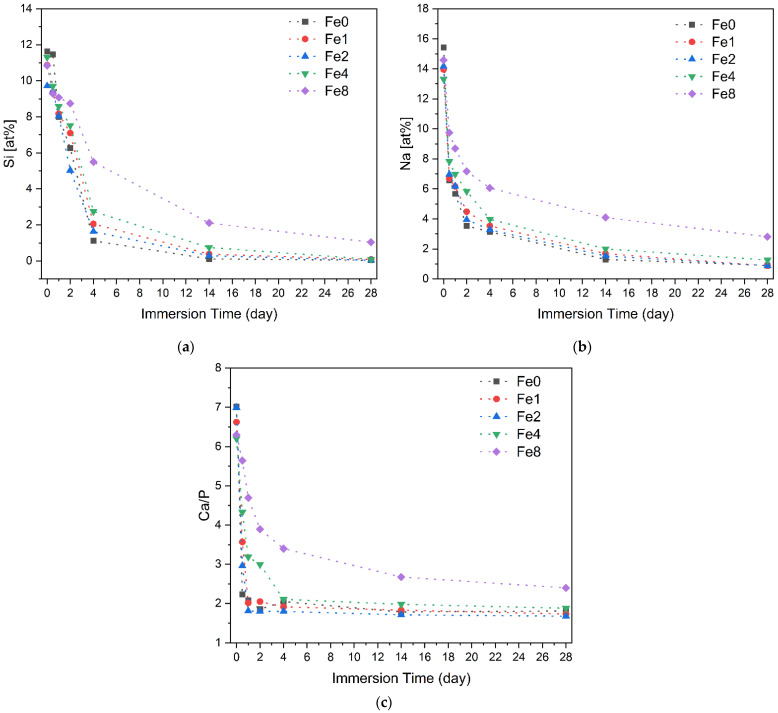
The variation in the atomic percentage of (**a**) Si and (**b**) Na elements and (**c**) the Ca/P ratio presented on the surface of the bioglass pellets after immersion in SBF.

To assess the kinetics of apatite precipitation on bioactive glass surfaces within a physiological environment, bioglass pellets underwent immersion in simulated body fluid (SBF) for varying durations: 12, 24, 48, 96 h, 14, and 28 days. SEM-EDS analysis was utilized to examine the atomic percentage of the elements presented on the surface. The Figure 5a,b reveals a gradual decline in silicon and sodium ions on the glass surface over time, reaching stabilization after 96 h. Indeed, the exposure of the bioglass to the SBF medium initiates ionic exchange between them. The pH rises as a consequence of the exchange of H^+^ and H_3_O^+^ ions in the medium with the alkali and alkaline earth ions (Na^+^ and Ca^2+^) on the bioglass surface. The breakage of Si-O-Si bonds in the glass network is facilitated by this rise in pH, which accelerates the glass’s dissolution and promotes the creation of silanol units (Si(OH)_4_). These silanol units condense onto the glass surface to create a layer of hydrated silica. This layer serves as a nucleation site for the subsequent crystallization of a carbonated hydroxyapatite layer [56,57]. 

**Figure 6:**
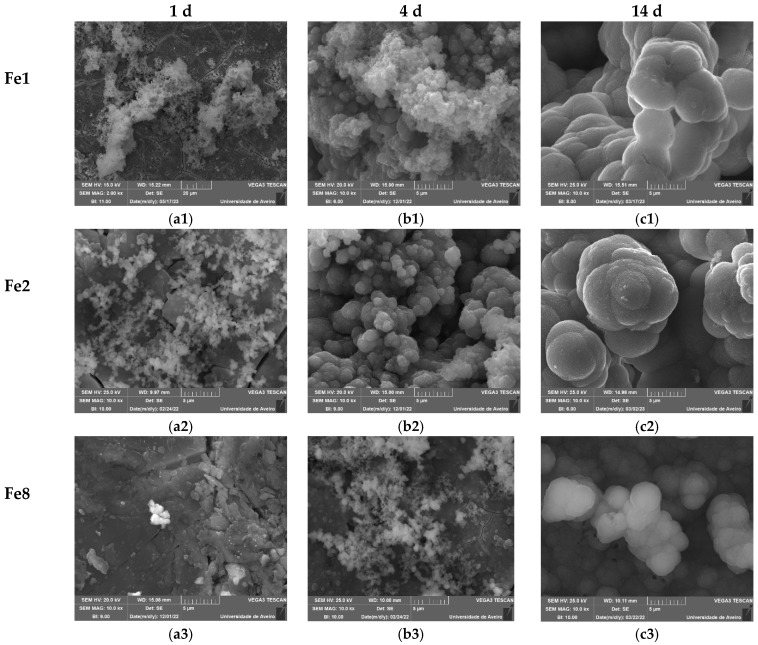
SEM micrographs of the surface of the bioglasses after immersion in SBF for (**a1**–**a3**) 1 d; (**b1**–**b3**) 4 d; (**c1**–**c3**) 14 d. (The magnification of SEM images is 10 kX).

Figure 5c illustrates the calcium-to-phosphorus (Ca/P) ratio, showing that for the samples with a low content of Fe_3_O_4_, this ratio decreases with increasing immersion time in SBF and stabilizes to reach a value close to that of hydroxyapatite in natural bone (Ca/P = 1.67) within the first 24 h [58]. However, for the samples with 8 mol% of Fe_3_O_4_, this value is considered high, reaching approximately 4 after 28 days of SBF immersion. SEM micrographs (Figure 6) validate the bioactivity of the glasses by revealing the presence of spherical (cauliflower-like) particles on the surfaces of the pellets. This observation provides evidence of the development of an apatitic layer. As immersion time in SBF increased, the apatite particles aggregated and became denser. After 14 days of immersion, this process resulted in the complete coating of the surface with an apatite layer. In the case of the bioglass modified with 8 mol% of Fe_3_O_4_, small amounts of apatite particles are observed during the initial days of immersion in SBF. After 14 days, the size of these particles is notably smaller compared to those found in other bioglass samples, indicating reduced bioactivity.

**Figure 7:**
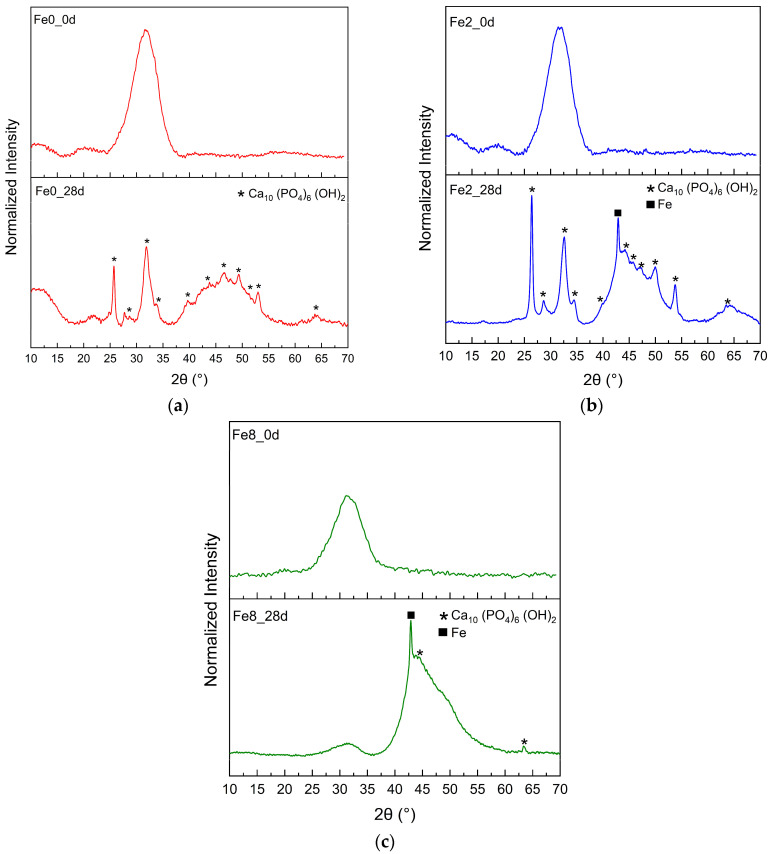
XRD patterns of (**a**) Fe0 (**b**) Fe2 and (**c**) Fe8 samples before (_0d) and after immersion in SBF for 28 d.

Figure 7 shows the XRD pattern of the Fe0, Fe2, and Fe3 samples before and after immersion in SBF for 28 d. Before immersion in SBF, the XRD patterns of the samples did not exhibit any distinct diffraction peaks but rather an amorphous hump, indicating a lack of long-range atomic order in their molecular arrangement. However, the immersion of the samples in SBF for 28 d led to the formation of a crystalline phase, with the main diffraction peak corresponding to hydroxyapatite Ca_10_ (PO_4_)_6_ (OH)_2_ (ICCD. No 00-001-1008) [59]. Moreover, the XRD results indicate that the apatite formed on the surface of bioglass with a high concentration of Fe_3_O_4_ exhibits a lower crystallinity than those formed on the samples with a low concentration of Fe_3_O_4_, suggesting that a high content of iron delays the crystallization of the apatite layer. These results are in agreement with the SEM analysis, which shows the formation of an apatite layer on the bioglass surface immersed in SBF. Additionally, the SEM analysis reveals that the Fe8 sample exhibits the smallest apatite particles on its surface compared to the other bioglass samples.

**Figure 8:**
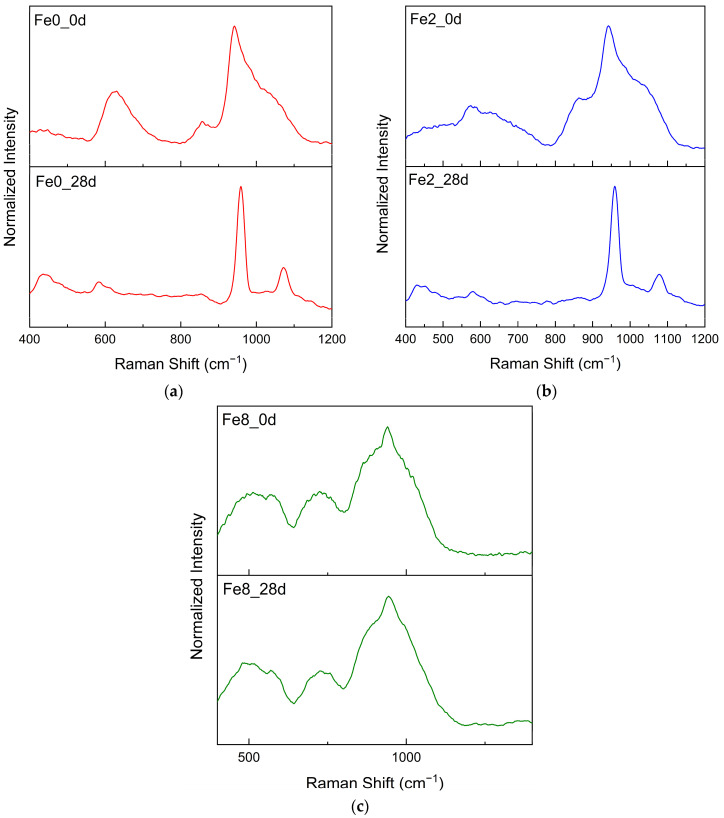
Raman spectra of (**a**) Fe0 (**b**) Fe2 and (**c**) Fe8 samples before (_0d) and after immersion in SBF for 28 d.

Figure 8 represents Raman spectra of bioglass samples before and after immersion of 28 d in SBF solution. The samples with low content of Fe_3_O_4_, after immersion in SBF for 28 d, show changes in their Raman spectra compared to their initial spectra, becoming very similar to that of commercial hydroxyapatite [60,61]. With SBF immersion, the bands attributed to the vibration of Si structural units at 800–1100 cm^−1^ become less intense, and the band associated with PO_4_ vibration becomes more pronounced and shifts to 960 cm^−1^. Moreover, additional bands related to PO_4_ vibration appeared at 440 and 580 cm^−1^. The band around 1080 cm^−1^ is associated with a carbonate group, indicating that the in vitro-grown hydroxyapatite is carbonated [61,62]. However, the Fe8 sample with a high Fe_3_O_4_ concentration did not reveal an alteration in Raman spectra. This result is consistent with the SEM and XRD findings, revealing that at high concentrations, Fe_3_O_4_ decreases the formation of the hydroxyapatite layer.

**Figure 9:**
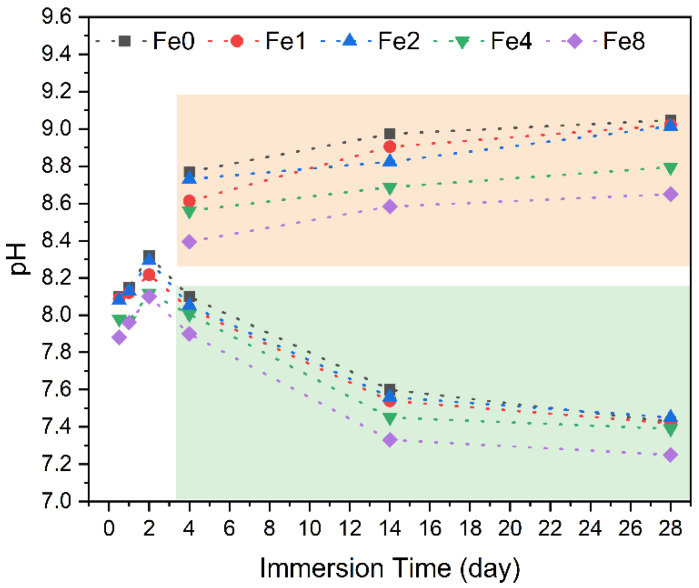
Variation in pH values of the SBF solution resulting from pellets immersion for different times, with the medium changed every two days (green rectangle) and without medium change (orange rectangle).

Figure 9 depicts the pH variations in SBF over different immersion times for pellets under two conditions: with regular medium changes every two days to mimic the behavior of the material in the physiological environment, and continuous exposure without medium replacement. As previously mentioned, the pH levels of the surrounding SBF solution in contact with the bioglass gradually increased over time due to the dissolution of alkaline metal ions (Na^+^ and Ca^2+^) from the bioglass pellets. Notably, during the initial two days, this increase was particularly prominent, with a more gradual rise observed during the subsequent period, as evidenced by the values enclosed within the orange rectangle (representing samples where the medium remained unchanged). However, when the immersion conditions were altered to mimic those of the in vivo environment, with medium changes every two days, a decrease in pH was observed (the values enclosed within the green rectangle). The decrease in pH suggests the formation of a hydroxyapatite layer on the bioglass surface [56,63].

### 3.4. Antibacterial Activity

**Figure 10:**
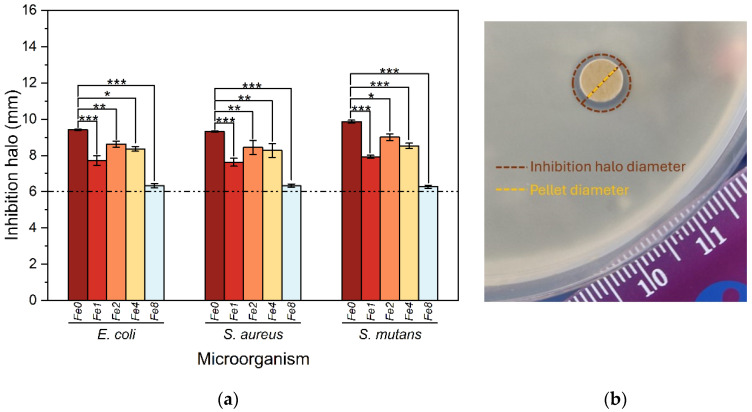
(**a**) Values of inhibition halo diameters of the bioglass samples modified with the insertion of various Fe_3_O_4_ concentrations against *E. coli*, *S. aureus*, and *S. mutans* bacteria after incubation for 24 h. Results are reported as mean ± SD. The asterisks indicate significance in an unpaired *t*-test; * *p* ≤ 0.05; ** *p* ≤ 0.01; *** *p* ≤ 0.001. (**b**) An example of an essay plate illustrating the inhibition halo of a Fe2 pellet on *E. coli*.

The antibacterial activity of the prepared bioglasses was assessed using the agar diffusion method against *E. coli*, *S. aureus*, and *S. mutans*. The inhibition halo diameters of the pellets against various bacteria are presented in Figure 10. 

The results obtained demonstrate the antibacterial efficacy of all samples, as evidenced by the presence of an inhibition zone surrounding the Bioglass pellets. The observed mean diameter of these zones, exceeding 6 mm, surpasses the diameter of the pellets, indicating substantial antibacterial activity against all tested bacterial strains. Two main processes elucidate the antibacterial properties of 45S5 bioglass. These processes involve the release of bioglass ions, particularly Na^+^ and Ca^2+^, which induce alkalinity in the surrounding medium, thereby increasing osmotic pressure [18,19]. This alkaline pH inhibits bacterial growth and metabolic processes by disrupting proteins and enzymes. Furthermore, the discharge of ions and subsequent fluctuations in their concentrations affect the integrity of bacterial cell membranes and intramembrane pressure, leading to changes in cellular size, shape, and membrane tension levels, ultimately resulting in bacterial death. A notable decrease in antibacterial efficacy was observed in samples modified with the insertion of Fe_3_O_4_ compared to the base material. This decline in antibacterial effect can be attributed to the presence of iron. This finding is supported by the analysis conducted to evaluate the pH levels of the SBF medium after immersion of the samples. Remarkably, the pH measurements show that samples containing Fe_3_O_4_ exhibited lower pH values compared to the base material, with higher concentrations of iron displaying minimal pH values. Therefore, the presence of iron in the modified samples contributes to the alteration of pH levels, which in turn could potentially influence their antibacterial properties. The bioglass modified with 2 mol% Fe_3_O_4_ showed a higher antibacterial activity compared to all modified bioglasses with Fe_3_O_4_. It exhibits a mean inhibition of halo diameter of 8.61 mm, 8.44 mm and 9.01 mm against *E. coli*, *S. aureus* and *S. mutans* bacteria, respectively.

## 4. Conclusions

Several bioglass samples modified with the addition of Fe_3_O_4_ were successfully produced using the melt-quenching technique. Structural analysis of the samples using Raman spectroscopy revealed that the insertion of Fe_3_O_4_ alters the bioglass structure, leading to the appearance of new vibrations related to the formation of Fe-related structural units. The bioactivity test showed that within 24 h, most samples formed a calcium phosphate-rich layer, except for the sample modified with 8 mol% of Fe_3_O_4_, which exhibited slower reactivity, developing this layer within 96 h. The antibacterial tests showed that the prepared glasses exhibited antibacterial activity against E. coli, S. aureus, and S. mutans bacteria. However, the modified bioglass with Fe_3_O_4_ did not exhibit notable antibacterial properties compared to the 45S5 bioglass. Among all modified bioglasses with Fe_3_O_4_ addition, the bioglass with 2 mol% Fe_3_O_4_ showed higher antibacterial properties.

## Figures and Tables

**Table 1:** The composition of different bioglass samples.

Composition (mol%)					
Sample	SiO_2_	Na_2_O	CaO	P_2_O_5_	Fe_3_O_4_
Fe0	46.10	24.40	26.90	2.60	-
Fe1	45.64	24.16	26.63	2.57	1
Fe2	45.18	23.91	26.36	2.55	2
Fe4	44.26	23.42	25.82	2.50	4
Fe8	42.41	22.45	24.75	2.39	8

## Data Availability

The data presented in this study are available from the corresponding author upon request.
